# Characterization of the robust humoral immune response to GSK2618960, a humanized anti-IL-7 receptor monoclonal antibody, observed in healthy subjects in a Phase 1 study

**DOI:** 10.1371/journal.pone.0249049

**Published:** 2021-03-23

**Authors:** Karen Liao, Keguan Chen, Sara Brett, Andrew Gehman, Ann M. Schwartz, George R. Gunn, Stephen L. DeWall

**Affiliations:** 1 Immunogenicity Group, GlaxoSmithKline, Collegeville, Pennsylvania, United States of America; 2 Oncology Cell Therapy, Oncology R&D, Stevenage, United Kingdom; 3 Research Statistics, GlaxoSmithKline, Collegeville, Pennsylvania, United States of America; Emory University School of Medicine, UNITED STATES

## Abstract

Interleukin-7 (IL-7) signaling modulates T cell activity and is implicated in numerous autoimmune diseases. An anti-IL-7 receptor monoclonal antibody (GSK2618960) biotherapeutic was evaluated in healthy subjects for safety, pharmacokinetics (PK), pharmacodynamics (PD) and immunogenicity in a single-dose escalation phase I study. We found that antibodies against GSK2618960 (i.e., anti-drug antibodies or ADA) developed in 83% and 100% of GSK2618960-treated subjects in the 0.6 and 2.0 mg/kg dose cohorts, respectively. Of the ADA positive subjects, 64% (7 of 11) had detectable neutralizing activity. Further investigation revealed the presence of GSK2618960-specific memory B cells, indicating the development of immunological memory for the ADAs. *Ex vivo* stimulation of peripheral blood mononuclear cell (PBMC) samples demonstrated a relatively strong CD4^+^ T cell proliferation response to GSK2618960 as compared to the control anti-RSV antibody (which is known to have only low immunogenic potential), confirming the high immunogenic potential of GSK2618960. Furthermore, GSK2618960 was found to bind *in vitro* monocyte-derived dendritic cells (DCs). GSK2618960 treatment of PBMCs increased the proportion of DC cells showing an increase in expression of CD83, CD86 and CD209, which indicated enhanced DC differentiation and activation relative to the isotype control anti-β amyloid antibody. Collectively, the evidence supports that the high incidence of observed clinical immunogenicity was likely related to the receptor-mediated activity by GSK2618960.

## 1. Introduction

Clinical immunogenicity data for approved therapeutic mAbs indicate that most humanized or human antibodies generally have a relatively lower risk of immunogenicity in both incidence and consequence compared to non-human and chimeric antibodies [1]. Of course there are exceptions, alemtuzumab, despite being humanized, induced anti-drug antibodies (ADAs) with neutralization activity detected in 30% to 70% of patients [2]. Adalimumab, a fully human monoclonal antibody (mAb) also demonstrated a relatively high incidence of immunogenicity, with ADAs and neutralizing antibodies detected in 5% to 89% patients depending on the disease and concomitant medications [3]. For another humanized antibody, bococizumab, ADAs developed in a large percentage of the patients, and higher titer responses correlated with faster drug clearance and reduced efficacy [4]. The causes of immune responses to biotherapeutics are multifactorial and still not fully understood. The risk factors that can contribute to immunogenicity have been classified as either extrinsic or intrinsic [5–7]. The extrinsic risk factors may be patient-related (e.g., immune status, genotype/HLA, etc.), product-related (e.g., aggregates and host-cell protein content), and treatment-related (e.g., dose, frequency of dose, route of administration). Intrinsic factors may include the primary sequence (e.g., non-human sequences pose increased immunogenic risk) and structural/chemical modifications to the biotherapeutic (e.g., pegylation). Another key factor that can contribute to the immunogenicity risk is the mechanism of action (MoA) of the biotherapeutic. The MoA may potentially lead to target-mediated biological effects that increase the immunogenic potential of the biotherapeutic. For example, immunomodulatory biotherapeutics (i.e., mAbs) that target T cells or antigen-presenting cells have increased risk for clinical immunogenicity compared to biotherapeutics that target B cells [8]. In general, target-mediated immunogenicity, believed to be related to the MoA of a biotherapeutic, has not been well characterized or understood, and, consequently, it remains difficult to predict even for biotherapeutics that target immune cells.

One such example of humanized mAb with high ADA incidence was presented in a recent report from a Phase 1 study (Study 200902, identifier: NCT02293161) [9]. Study 200902 consisted of two cohorts of healthy volunteers who were administered either placebo or GSK2618960 (0.6 or 2.0 mg/kg) as a single intravenous (IV) dose. From this study, 83% (5 of 6) of GSK261896-treated subjects in the 0.6 mg/kg cohort and 100% (all 6) of GSK261896-treated subjects in the 2.0 mg/kg cohort were confirmed positive for anti-GSK2618960 antibodies. Additionally, most subjects (64%) that confirmed positive for anti-GSK2618960 antibodies also had neutralizing activity. Of note, no safety event appeared to be associated with the immunogenicity.

GSK2618960 is a humanized, Fc-disabled immunoglobulin G1 (IgG1) mAb that binds to the α-chain of the heterodimeric IL-7R to block IL-7 binding and intracellular signaling. There is increasing evidence which implicates aberrant IL-7/IL-7R signaling in the pathogenesis of numerous autoimmune diseases, such as rheumatoid arthritis [10, 11], type I diabetes [12, 13], MS [14, 15], systemic lupus erythematosus [16] and Primary Sjögren’s Syndrome [17–19]. Therefore, targeting components in IL-7 signaling pathway may be of therapeutic benefit for these autoimmune diseases. IL-7 is a lymphopoietic cytokine that is essential for early lymphocyte development, and it is also a modulator of peripheral T cell homeostasis [20–22]. The receptor of IL-7 (i.e., IL-7R) consists of two subunits: the IL-7 ligand-binding chain (IL-7Rα; CD127) and the common γ-signaling chain (γc; CD132) [23]. Signaling through IL-7R upon binding of IL-7 promotes survival, proliferation and differentiation of T cells [24, 25]. The IL-7Rα chain is expressed on naïve and memory CD4^+^ and CD8^+^ T cells [24–26], while expression is notably low on FOXP3^+^ regulatory T (Treg) cells [27]. Additionally, the IL-7Rα chain can also form a heterodimer with the thymic stromal lymphopoietin receptor (TSLPR) to bind thymic stromal lymphopoietin (TSLP). TSLP, a member of the IL-7 family of cytokines, functions mainly on myeloid cells, and it plays a role in the regulation of dendritic cells (DCs) [28]. Signaling via TSLPR and IL-7Rα enhances the maturation of CD11c^+^ DCs and induces CD11c^+^ DC-primed CD4^+^ T cell proliferation [29–32]. TSLP has been shown to activate CD11c^+^ DC *ex vivo* and direct the differentiation of T helper 2 (Th2), indicating a potential role of DCs in allergic inflammation [33].

## 2. Materials and methods

### 2.1. Clinical study, serum and PBMC samples

The GSK clinical study (200902) was a phase I randomized, double-blind (sponsor-unblind), placebo-controlled study performed in a single center in Cambridge, UK, between November 2014 and September 2015. The study was conducted in accordance with Good Clinical Practice and the Declaration of Helsinki 2013, and the local regulations. The protocol was approved by the local ethics committee (14/LO/1670, National Research Ethics Service Committee, Lodon, UK) and all study subjects provided written informed consent. The study was registered on Clinicaltrials.gov (identifier: NCT02293161). Subject inclusion and exclusion criteria can be found in clinical protocol in S1 File. Two single ascending dose cohorts (0.6 mg/kg and 2 mg/kg), each with nine healthy subjects, were sequentially randomised (2:1) to receive either GSK2618960 or placebo as a single IV infusion (S1 Fig). Demographic details for the 18 participating subjects are reported previously [9]. Serum samples were collected at Day -1 (pre-dose), Day 15, Day 22 (2 mg/kg cohort only), Day 29, Day 85, and Day 169 for immunogenicity assessment. Heparinized whole blood was collected at Day -1 (pre-dose), Day 29, Day 43, and Day 169, and PBMCs were isolated by Ficoll–Hypaque density gradient centrifugation and frozen in liquid nitrogen until use. Whole blood from healthy non-study subjects were obtained from Biological Specialty company (Colmar, PA), and PBMCs were isolated with same method as used for study subject PBMC isolation.

### 2.2. Antibodies and reagents

GSK2618960 and the Fc-control mAb [GSK933776, an anti-β-amyloid (BAM) mAb] were manufactured by GSK. Both of these therapeutic mAbs were clinical grade material produced in Chinese Hamster Ovary (CHO) cell lines under Good Manufacturing Practices (for sequences and manufacture process, refer to US patent aplication US13/574,847 in which referred to as 1A11 H3L4 for GSK2618960 and US14/881,428 referred to as H2L1 for GSK933776, respectively) and both had acceptable product quality attributes (% aggregates, host cell protein content, glycosylation, etc…) based on manufacturing specifications. The negative control mAb, a humanized anti-Respiratory Syncytial Virus (anti-RSV mAb), with reported low clinical immunogenicity incidence was purchased from AbbVie (Worcester, MA). Biotin (Pierce, Waltham, MA) and ruthenium (Gold Sulfo Tag NHS-Ester, Meso Scale Discovery, Rockville, MD) labelled GSK2618960 conjugates were produced according to the manufacturer’s instructions (with challenge ratio of 12:1 for both conjugates). Recombinant human IL-7R extracellular domain Fc-fusion protein (MW: 104 kDa) was produced by GSK, and ruthenium labelled with a challenge ratios of 12:1, according to the manufacturer’s instructions. The mouse anti-human IL-7Rα mAb (Clone 6A3) (i.e., target-blocking antibody) used in the ADA assay, NAb assay, and B-cell ELISPOT assay to reduce interference from the soluble form of IL-7Rα that may be present in the samples was produced by GSK. Streptavidin-coated 96-well plates and Read Buffer T were obtained from Meso Scale Discovery (MSD) (Rockville, MD). The rabbit anti-GSK2618960 polyclonal antibody used as the positive control for the ADA assay was affinity purified from sera from rabbits hyperimmunized with GSK2618960 (Covance, Chantilly, VA). The mouse anti-idiotype Fab against GSK2618960 used as positive control for NAb assay was generated by Bio-Rad (Puchheim, Germany). The negative control (NC) was pooled normal human sera sourced from BioIVT (Westbury, NY).

Test-B™ cell culture medium and B cell polyclonal stimulating reagent (B-poly-S), and B cell ELISPOT kit including Ig capture antibody, total IgG detection antibody, buffer and PVDF 96-well plates were purchased from CTL Immunospot (Shaker Heights, OH). X-VIVO^TM^ 20 medium was obtained from Lonza (Walkersville, MD). Human AB serum and recombinant hIL-2 were purchased from Sigma-Aldrich (St. Louis, MO). CD3 (APC-cy7, PerCP), CD4 (PerCpCy5.5, FITC), CD8 (PE), CD11c (APC), HLA-DR (BV605), CD40 (FITC), CD83 (BV510), CD86 (PE), CD209 (BV421) antibodies were all bought from BD Biosciences (San Jose, CA). Cell Trace Violet 450 and Viability dye L10119 were obtained from Invitrogen (Carlsbad, CA).

### 2.3. Anti-GSK2618960 antibody assay

Anti-GSK2618960 antibodies (ADAs) were detected using a homogenous electrochemiluminescent (ECL) bridging assay format, consisting of screening, confirmation, titration assays. These assays were validated for clinical sample testing according to regulatory guidelines and scientific recommendations [10]. Clinical samples were analyzed for anti-GSK2618960 antibodies following a tiered-testing scheme (i.e., screening, confirmation, and titration) [10]. First, all serum samples were tested in the screening assay. Briefly, serum samples were diluted 1:10 with buffer, then incubated with biotinylated and ruthenylated GSK2618960 (final concentrations: 0.5 μg/mL for biotinylated and 1.0 μg/mL for ruthenylated GSK2618960) for 16 to 20 hours (i.e., overnight) at room temperature under gentle shaking. After incubation, the mixture of sample and conjugates (50 μL/well) were transferred to a pre-blocked streptavidin-coated plate (MSD, Rockville, MD). Next, the plate was incubated for 1 hour at room temperature with gentle shaking. The plates were then washed with PBS containing 0.1% Tween-20 (PBST) followed by addition of 2x Read Buffer T. Finally, the plates were read on a MSD Sector® Imager 6000 Reader. The intensity of the ECL signal is directly proportional to the amount of anti-GSK2618960 antibodies in the sample. For the data analysis, the ECL signal of test sample was normalized to the signal of the negative control on the plate. The normalized result is referred to as relative ECL (RECL). From the validation, the statistically-derived screening assay cut point was 1.16 RECL (5% false-positive rate following standard practice) using a panel of normal (i.e., healthy) donor sera (N = 50) [11].

Samples that tested above the screening cut point were considered potentially positive, and then tested in the confirmation assay to determine the specificity of the assay signal by inhibition with free GSK2618960. Briefly, samples were 1:10 diluted with buffer containing the target-blocking antibody (anti-human IL-7Rα mAb; GSK clone 6A3 at 45 μg/mL). Soluble IL-7Rα has been reported previously [9, 12, 13]. To eliminate potential false positives from target interference, a target-blocking antibody that competes with GSK2618960 for binding to the soluble version of IL-7Rα was added in the confirmation assay. The diluted samples were first incubated with and without excess free drug GSK2618960 (101 μg/mL) for 1 hour and then proceeded for ADA detection as described in the screening assay. After reading the plate, the signal of the sample spiked with free GSK2618960 was compared to the signal of the unspiked sample by calculating the percent (%) inhibition. A signal decrease of 43.5% (validated confirmation assay cut point with 1% false positive rate) in the confirmation assay was deemed a GSK2618960-specific ADA response [14]. Finally, all confirmed positive samples were titrated in a validated titration assay to obtain ADA titer values. The samples were titrated by serial 2-fold dilutions (1:2) in negative control serum and tested in the ADA assay. Titer was reported as the reciprocal of the dilution at which the sample’s response (RECL) was just above the screening cut point.

### 2.4. Competitive-ligand-binding neutralizing anti-GSK2618960 antibody assay

To characterize the potential neutralizing activity of the anti-GSK2618960 antibodies, a validated competitive ligand binding assay was used to evaluate neutralization activity in confirmed positive ADA samples. Briefly, samples were diluted with dilution buffer containing the target-blocking antibody (anti-IL-7Rα mAb, GSK clone 6A3 at 50 μg/mL), and then mixed with biotinylated GSK2618960 (at final concentration of 0.125 μg/mL). The mixture was incubated overnight at room temperature with gentle shaking. After incubation, the sample and biotinylated GSK2618960 solution was transferred to a blocked MSD streptavidin-coated plate plate and allowed to bind for 1 hour with shaking. The MSD plate was washed and then 50 μL/well of ruthenium labelled IL-7Rα (IL-7Rα Fc fusion protein) was added. Next, the plate was incubated for 1 hour at room temperature. The MSD plate was washed and 2x Read Buffer T was added. The plate was read using MSD Sector® Imager 6000. A percentage (%) inhibition for each sample was calculated by the formula: 100 x (mean ECL of negative control–mean ECL of sample)/mean ECL of negative control. A sample with % inhibition greater than 11.4% (cut point established from healthy donors) was reported as positive for neutralizing antibody activity.

### 2.5. B cell ELISPOT assay for detection of GSK2618960-specific memory B cells

The B cell ELISPOT method for detection of biotherapeutic-specific memory B cells was previously described [15]. Briefly, PBMCs from study subjects were plated in 6-well plate at 2x10^6^/mL in Test-B™ Cell Culture Medium (CTL Immunospot) supplemented with polyclonal stimulator (B-poly-S from CTL Immunospot) for 7 days incubation at 37°C incubator with 5% CO_2_. Stimulated PBMCs were harvested and seeded in 96 well PVDF membrane plate at either 1x10^6^/well with 4 to 6 replicate wells (depending on cell recovery post-stimulation) for drug specific memory B cell enumeration, or duplicate wells at 2.5x10^4^ /well for total IgG detection. The 96 well PVDF plate was incubated at 37°C for overnight. Secreted antibodies from activated memory B cells were captured by anti-human IgG. Total IgG and GSK2618960 specific IgG were detected by biotin labelled anti-human IgG Fc and biotin labelled GSK2618960, respectively. Plate was scanned on ImmunoSpot Analyzers (Cellular Technology Limited, OH, US). Total IgG spots were counted with instrument software and GSK2618960 specific spots were counted manually due to the low frequency of specific spots. The average antibody secreting cell (ASC) spots from replicate wells was reported as memory B cell frequency (ASC events per million cells) for both total and GSK2618960-specific memory B cell activity. Total IgG ASC served as control to monitor memory B cell activation, a minimum 5000 total IgG spots per million cells were required for data inclusion.

### 2.6. *Ex vivo* T cell proliferation assay with PBMC from study subjects

To assess the immunogenic potential of GSK2618960, PBMC samples collected from study subjects were incubated *ex vivo* with either GSK2618960 or a control anti-RSV mAb to test and compare the ability of these mAbs to stimulate CD4^+^ T cells. The anti-RSV mAb was used as a control because of the reported low incidence of immunogenicity in the clinic [16]. Briefly, frozen cells were thawed and labelled with Cell Trace Violet (CTV). The labelled cells were resuspended with X-VIVO™ 20 medium supplemented with 2% heat inactivated human AB serum and 1 ng/mL recombinant human IL-2 to a density of 2x10^6^/mL, seeded at 150 μL per well in 96 well plate and incubated in a 37°C 5% CO_2_ incubator for overnight. Then GSK2618960 and control anti-RSV mAb were added to cell plates to final concentrations of 10 μg/mL (for GSK2618960 only) and 100 μg/mL (for both GSK2618960 and anti-RSV mAb). Cells were further incubated for 7 days at 37°C with 5% CO_2_. On day 7, the cell plates were centrifuged, and 100 μL of supernatant was removed, and replaced with fresh X-VIVO™ 20 medium containing 2% human AB serum and 1 ng/mL IL-2, and continued incubation until day 10. On day 10, cells were harvested and stained with cell surface markers for CD3 (PerCP), CD4 (FITC), CD8 (PE), and cell viability dye (F-NIR-A). Samples were acquired on BD FACSCanto 2 (BD Bioscience, San Jose CA). Events were cleaned by gating out dead cells and doublets. CTV dim CD3^+^ and CD4^+^ positive cells were defined as proliferating CD4^+^ T cells and expressed as percentage (%) of total CD4^+^ T cells.

### 2.7. Generation of monocyte-derived dendritic cells, GSK2618960 binding to DCs, and activation of DCs by GSK2618960

CD14^+^ monocytes were isolated by CD14 beads (Miltenyi, San Diego, CA) from freshly thawed PBMC. Purified monocytes were cultured in serum free medium (CellGenix, Freiburg, Germany) containing IL-4 (250 IU/mL) and GM-CSF (800 IU/mL) at density of 1x10^6^/mL for 5 days. On day 5, cells were collected, washed, and stained with anti-CD3, CD14, CD20, CD56, CD11c, anti-HLA-DR and biotin-labelled GSK2618960 or biotin-labelled Fc-control antibody (anti-BAM mAb). After 30 min RT incubation, samples were washed and stained with PE-conjugated streptavidin for 15 min incubation at RT. Samples were washed and read on a BD FACSCanto 10 (BD Bioscience, San Jose CA). The binding of GSK2618960 with freshly thawed PBMCs were conducted with the same method.

DC activation was evaluated after treatment with medium, GSK2618960, or Fc-control anti-β amyloid mAb using PBMCs from normal donors obtained from Biological Specialty Company (Colmar, PA). Briefly, 5 μg of GSK2618960 or Fc-control antibody were dry coated on a 12-well plate (equal volume of PBS was added for the media control wells). Next day, PBMCs were thawed and seeded on the antibody-coated wells at density of 4x10^6^ cells/mL in αMEM containing 5% AB serum, then treatment solutions of GSK2618960, or Fc control antibody or medium were added to cells to final concentration of 50 μg/mL in accordance with the mapping of antibody dry coating. Incubated cells in 37°C incubator with 5% CO_2_ for 4 days. At day 4 1mL medium was removed from each well and replenished with fresh medium containing 50 μg/mL of GSK2618960 or Fc-control antibody (equal volume of medium added to medium control wells), continued cell incubation for 3 days. On day 8 cells were collected and stained with antibody cocktail containing CD3 (APC-cy7), CD4 (PerCpCy5.5), CD11c (APC), HLA-DR (BV605), CD40 (FITC), CD83 (BV510), CD86 (PE), CD209 (BV421). Samples were acquired on the BD FACSCanto 10 (BD Bioscience, San Jose CA). Events were cleaned by gating out dead cells and doublets, and DC subset was defined as CD3^-^/CD11c^+^ and HLA-DR^+^. Expressions of CD40, CD83, CD86, and CD209 on DC subset were compared between treatments within same donor.

### 2.8. Statistical analysis

Association of two incidence measures (e.g., treatment with GSK2618960 and positive response for memory B cells) was tested using Fisher’s exact test due to small sample sizes [17]. Spearman’s rank correlation coefficient was used for correlation analysis when discrete, ordered measures with ties, such as ADA titer, were involved [18]. Memory B cell frequency data, which exhibited ties and a substantial number of zero values, was fit with a generalized linear mixed-effect model (exposure group as a fixed effect, donor as a random effect, and a Poisson distribution), with pairwise comparisons by exposure group. Mixed-effect models were fit to the log-transformed *ex vivo* CD4 proliferation data. In the first model, fixed effects were dose group, study day, treatment, and all 2- and 3-way interactions; and random effects were donor and cohort. In the second model, *in vivo* drug exposure group was included instead of dose group. In both models, 3- or 2- way interaction terms involving treatment which were highly insignificant (p-value > 0.50, indicating a consistent relative impact of treatments) were dropped, and pairwise comparisons of treatments on (geometric) mean stimulation index (SI) averaging across dose group and study day (first model) or by exposure group averaging across study day (second model) were conducted. For the analysis of % DC subset, the log-transformed data was fit to a two-way ANOVA model with terms for donor, treatment, and their interaction. Then, treatment group (geometric) means were compared within each donor. All statistical tests were conducted without adjusting for multiplicity due to the exploratory nature of this study. Conclusions of any difference or effect were based not only on “statistical significance” with p-value < 0.05, but also using error bars (standard error and confidence intervals) to display the effect size as part of the data interpretation.

## 3. Results

### 3.1. High incidence of ADA was observed post single dose of GSK2618960

**Fig 1:**
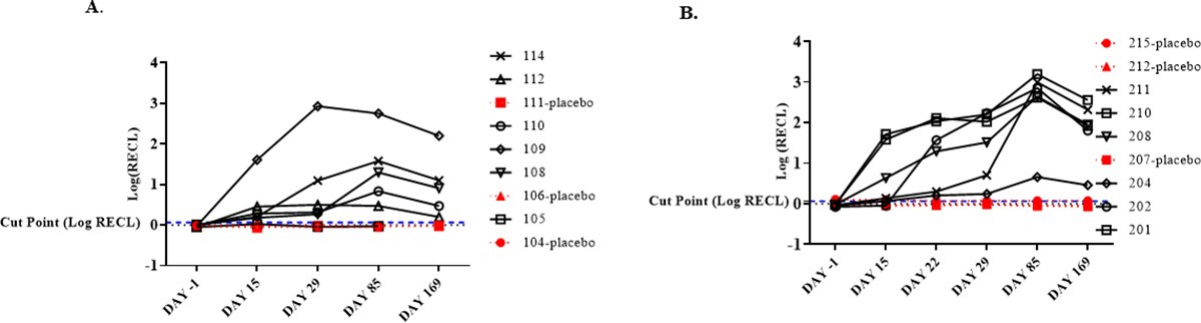
ADA responses observed in the phase I study 200902. Assay signal (ECL) was normalized to negative control and plotted as logRECL. The blue dotted line represents assay cut point (1.16 RECL). (A). Cohort A (0.6 mg/kg dosing group). (B). Cohort B (2 mg/kg dosing group). Each cohort had 9 subjects with 6 subjects on treatment and 3 on placebo.

For ADA evaluation, serum samples were collected at Day -1 (pre-dose) and post-dose at Day 15, Day 22 (cohort B only), Day 29, Day 85, and Day 169 (follow-up). The time course of ADA responses for study subjects are displayed in Fig 1. Five (5) of the six (6) treated subjects (83%) in the 0.6 mg/kg dose group (Fig 1A), and all six (6) treated subjects (100%) in the 2 mg/kg group (Fig 1B) developed ADAs. All subjects that received the placebo tested ADA negative. ADAs were detected at the earliest sampling time (Day 15) in 6 of the 11 positive subjects, and the remaining subjects developed ADAs at either Day 22 or Day 29. Ten (10) out of the 11 positive responders remained ADA positive at the last time point (Day 169), suggesting a persistent ADA response [19, 20]. Subjects from cohort B showed generally higher titers compared to cohort A (Table 1), indicating a stronger ADA response with increased dose. The treatment-induced ADAs were also characterized for neutralizing activity in a validated NAb assay. As shown in Table 1. NAb activity was detected in 2 of 5 ADA-positive subjects (40%) in the 0.6 mg/kg cohort and 5 of 6 (83%) ADA-positive subjects in the 2.0 mg/kg cohort.

**Table 1:** Summary of ADA titer and NAb result for ADA positive response subjects.

Assay type	Time (day)	Cohort A (0.6 mg/kg)					Cohort B (2.0 mg/kg)					
		subj 108	subj 109	subj 110	subj 112	subj 114	subj 201	subj 202	Subj 204	subj 208	subj 210	subj 211
ADA	-1 (pre-dose)	nd	nd	nd	nd	nd	nd	nd	nd	nd	nd	nd
	15	nd	256	8	8	nd	320	nd	nd	32	160	nd
	22*	n/a *	n/a	n/a	n/a	n/a	640	320	4	80	640	8
	29	4	2560	4	8	80	320	320	2	160	320	16
	85	40	1600	32	4	160	5120	1280	16	1280	1280	2560
	169	40	1024	16	neg	64	2560	320	16	640	640	1280
NAb	85	neg	pos	neg	neg	pos	pos	pos	neg	pos	pos	pos
	169	neg	pos	neg	neg	neg	pos	pos	neg	pos	pos	pos

nd: titer not determined (ADA negative samples)

n/a *: Day 22 samples were not collected for cohort A.

In summary, GSK2618960 was observed to induce a strong humoral response in 92% (11 of 12) of healthy subjects treated with a single dose of GSK2618960. The ADAs appeared to be dose-dependent, as higher incidence, higher titer, and increased percentage of subjects with neutralizing ADA were associated with the 2 mg/kgGSK2618960 dose cohort. Of note, the development of ADAs was not observed to be associated with any safety events [9].

### 3.2. Memory B cells against GSK2618960 were detected following single dose in study subjects

**Fig 2:**
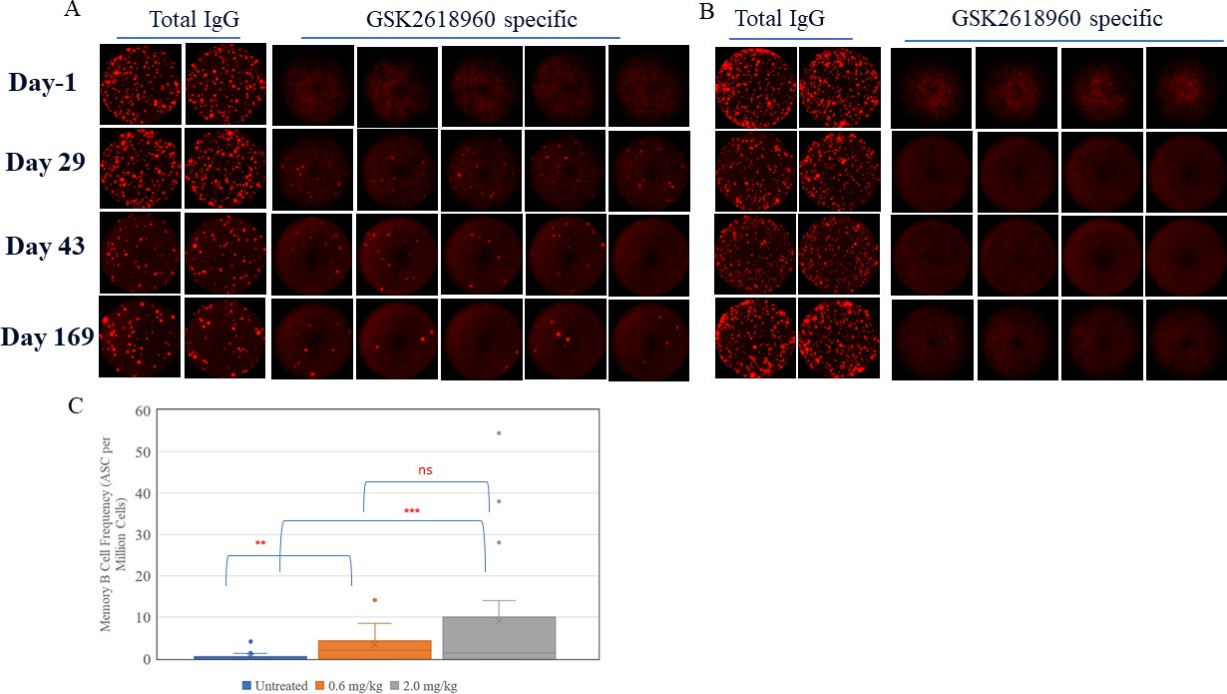
ADA specific memory B cell detection—Representative B cell ELISPOT images and distribution of memory B cell frequencies. (A). Subject #202 –post-GSK2618960 treatment 2mg/kg, ADA positive. (B). Subject # 207, placebo, ADA negative. (C). Distribution of memory B cell frequency from untreated (all sample time points for placebo subjects and Day-1 from treated subjects), 0.6 mg/kg dose cohort, and 2.0 mg/kg dose cohort samples. Statistical comparison test results: ‘ns’ = not statistically significant (p-value ≥ 0.050); ‘*’ = p-value < 0.050; ‘**’ = p-value < 0.010; ‘***’ = p-value < 0.001.

The data presented above showed that GSK2618960 was highly immunogenic in healthy human subjects after a single dose. To further characterize this robust humoral response, PBMC samples from the study subjects were evaluated to determine whether the ADA-positive subjects had developed memory B cell immunity. Using a B-cell ELISPOT assay, samples from 17 subjects across both treatment cohorts, with either three (Day -1, 29, and Day 169 for 0.6 mg/kg cohort) or four time points (Day -1, 29, 43, and Day 169 for 2 mg/kg cohort) per subject were evaluated. Representative B cell ELISPOT data from either GSK2618960-treated or untreated (placebo) subjects were shown in Fig 2. As shown in Fig 2A & 2B, robust total IgG ASCs were observed across all four time points for both subjects, indicating a robust memory B cell activation. The dosed subject which was ADA positive post-dose showed clear GSK2618960-specific ASC spots at all three post-dose time points (Day 29, Day 43, and Day 169), and the specific spots were not seen in the pre-dose sample (Day -1) (Fig 2A). In contrast, a subject which was administered placebo and was ADA negative, did not show GSK2618960-specific ASCs at any time points (Fig 2B). The GSK2618960-specific memory B cell responses by memory B cell frequencies (ASC per million cells) were compared by the drug treatment (i.e. drug treated or untreated) (Fig 2C). GSK2618960-specific memory B cell frequencies were undetectable in most of pre-dose or placebo samples (22 of 32 samples). The few untreated samples with detectable events had low frequencies which may be attributable to assay background (S1 Table). Post-GSK2618960 treatment, both cohorts demonstrated significantly higher GSK2618960 memory B cell frequencies compared to untreated samples (p<0.01 for 0.6 mg/kg cohort; p<0.001 for 2.0 mg/kg cohort). While the 2mg/kg treatment group seemed to have higher specific memory frequencies than 0.6 mg/kg group, the difference between the two dose cohorts was not significant (Fig 2C). A sample with 2-fold increase of ASCs compared to pre-dose (Day -1) sample from the same subject was considered positive for memory B cell activity. Furthermore, a subject with one or more time points that were positive for memory B cell activity was considered to have a positive memory B cell response. Applying these criteria, 91% (10 of 11) treated subjects demonstrated GSK2618960-specific memory B cell activity with average frequency of 12.2 ASC per million cells across all positive donors. In contrast, one out of six subjects (17%) on placebo showed a positive response with average ASC of 1.7 (S1 Table).

**Fig 3:**
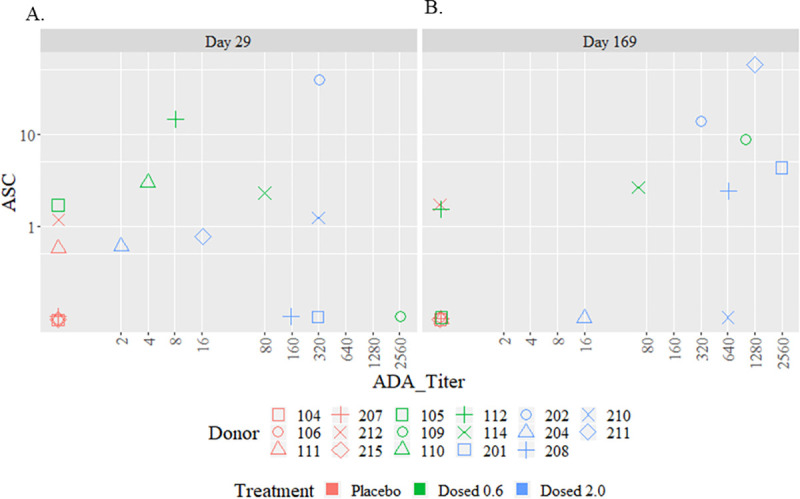
Correlation between ADA titer and memory B activity (mean ASC spots per million cells). (A). Day 29 (Spearman coefficient = 0.19; p-value = 0.459. (B). Day 169 with Spearman coefficient = 0.69; p-value = 0.002. In summary, the observed GSK2618960-specific ASC events in PBMC samples from study subjects were related to GSK2618960 treatment, indicating the development of immune memory activity for anti-GSK2618960 antibody response.

Statistical analyses were conducted to examine the correlation of GSK2618960-specific memory B cells with GSK2618960 treatment or ADA status. This revealed that GSK2618960-specific B memory responses were associated with both GSK2618960 treatment (p-value = 0.005) and the ADA status (p-value = 0.035) (Table 2). These results provide evidence of a positive relationship between memory B cell activity with GSK2618960 treatment and ADA status. Consequently, we evaluated whether the ADA titers correlated with memory B cell intensity (in ASC frequency per million cells). The memory B cell activity (mean ASCs frequencies from replicate wells) and corresponding ADA titer at post-treatment time points for the 17 subjects were analyzed statistically for potential correlation. Correlation analysis between ADA titer and memory B activity (mean ASC spots per million cells) were performed with samples grouped by time points for samples collected on Day 29 and Day 169. The Day -1 and Day 43 data were not included in this analysis because there were either no ADA positive samples (Day -1) or insufficient number of samples were available (Day 43). The analysis found no evidence of correlation on Day 29 (Spearman coefficient = 0.19; p-value = 0.459). There was evidence of a semi-quantitative correlation between ADA titer and memory B activity on Day 169 (Spearman coefficient = 0.69; p-value = 0.002) (Fig 3), suggesting the strength of memory B cell activity (measured by ASC frequency per million cells) correlated with ADA titer at the Day 169 time point.

**Table 2:** Correlation of GSK2618960-specific memory B cells with GSK2618960 treatment.

p-value = 0.005		**Memory B Cell Status**	
		Negative	Positive
**GSK2618960 Treatment**	No	**5**	**1**
	Yes	**1**	**10**
p-value = 0.035		**Memory B Cell Status**	
		Negative	Positive
**GSK2618960 ADA Status**	Negative	**5**	**2**
	Positive	**1**	**9**

### 3.3. *Ex vivo* CD4^+^ T cell proliferation by GSK2618960

**Fig 4:**
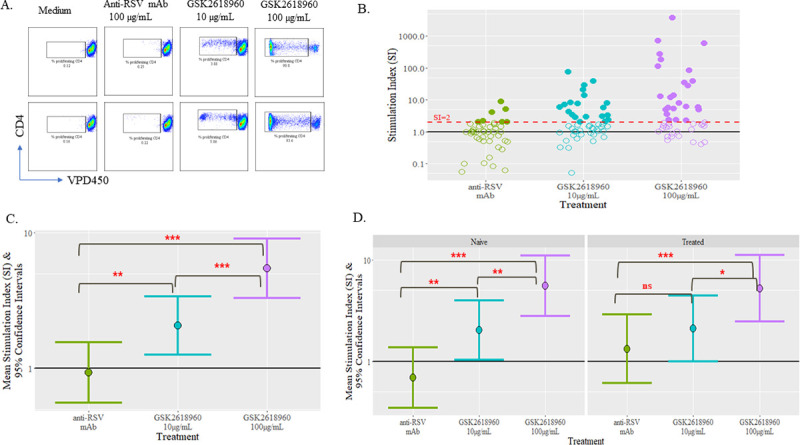
CD4^+^ T cell proliferation in response to GSK2618960 and control anti-RSV antibody. PBMCs collected from study subjects were treated with GSK2816960 (10 μg/mL and 100 μg/mL) and anti-RSV mAb (100 μg/mL); medium control was included for each sample. After 10 days of incubation, proliferating CD4^+^ T cells were determined by dilution of Violet Proliferation Dye (VPD450). Stimulation index (SI) was calculated for each treatment by formula SI = mean % of proliferating CD4 T cells in presence of GSK2618960 or anti-RSV / mean % of proliferating CD4 T in medium controls of same sample. (A). Representative flow cytometry plots of CD4^+^ T proliferation in responses to medium, control anti-RSV mAb, GSK2618960 at 10 μg/mL or 100 μg/mL concentration. (B). CD4^+^ T cell proliferation responses by SI to GSK2618960 or control anti-RSV mAb. Open circles are samples with SI<2. (C). Mixed-model mean SI and 95% confidence intervals in responses to control anti-RSV mAb, and GSK2618960 treatment. (D). Comparison of responses of GSK2618960 in naïve and treated samples. Statistical comparison test results: ‘ns’ = not statistically significant (p-value ≥ 0.050); ‘*’ = p-value < 0.050; ‘**’ = p-value < 0.010; ‘***’ = p-value < 0.001.

To further understand the high incidence of ADA observed in this clinical study, PBMC samples collected from the study subjects were stimulated *ex vivo* with GSK2618960 to determine the T cell activation potential of this compound. All 9 subjects from 0.6 mg/kg dosing cohort and 8 of 9 subjects from 2.0 mg/kg cohort were included in this evaluation, with three time points (Day -1, Day 29, and Day 169) evaluated for each subject. Each PBMC sample was incubated with either cell culture medium, control anti-RSV mAb (100 μg/mL), or two concentrations of GSK2618960 (10 μg/mL and 100 μg/mL) for 10 days. CD4^+^ T cell proliferation was measured by CTV dilution. Representative CD4^+^ T proliferation in responses to cell culture medium, control anti-RSV mAb, and 10 μg/mL or 100 μg/mL GSK2618960 at are shown in Fig 4A. The degree of CD4^+^ T cell stimulation in response to control anti-RSV mAb or GSK2618960 was measured by stimulation index (SI). The SI was the ratio of the mean percentage of proliferating CD4^+^ T cells from replicate wells of treatment to the mean percentage of proliferating CD4^+^ T cells from medium controls for the same sample.

Responses to control anti-RSV mAb, and 10 μg/mL or 100 μg/mL GSK2618960 treatment (in SI) are shown in Fig 4B. Comparing to the control anti-RSV mAb, GSK2618960 demonstrated more robust CD4^+^ T cell proliferation as indicated by a greater number of samples with SI above the empirical threshold (SI = 2) (Fig 4B). Statistical analysis indicated that CD4^+^ T cell proliferation responses at 10 μg/mL and 100 μg/mL GSK2618960 treatment were significantly higher compared to the control antibody, regardless of study day or dose group (Fig 4C). The 10 μg/mL of GSK2618960 treatment showed overall weaker stimulation compared to 100 μg/mL treatment, suggesting that CD4^+^ T cell stimulation by GSK2618960 may be dose dependent.

Since we observed memory B cell activity for ADA-positive subjects, we wanted to specifically explore whether *in vivo* drug exposure had any impact on the *ex vivo* potentiation of GSK2618960 on CD4^+^ T cell proliferation. To explore the potential impact, the data was examined by whether the PBMC samples were exposed to GSK2618960 *in vivo*. The untreated group (or naive) included samples from placebo group and pre-dose samples from treated subjects. The treated samples were PBMC samples collected post GSK261896-dosing at Day 29 and Day 169. The overall responses to GSK2618960 and control mAb were similar in GSK2618960-naive and dosed groups; and GSK2618960 treatment, particularly, at 100 μg/mL, demonstrated stronger CD4 T cell proliferation compared to control antibody in both groups (Fig 4D). The observations that *in vivo* exposure to GSK2618960 had no apparent impact on *ex vivo* potentiation of CD4 T cells and that the CD4 T cells proliferated in drug naïve samples indicated that the observed CD4 T cell proliferation was likely due to naïve CD4 T cell activation by GSK2618960 under these *ex vivo* treatment conditions.

### 3.4. Binding and activation of dendritic cells by GSK2618960

**Fig 5:**
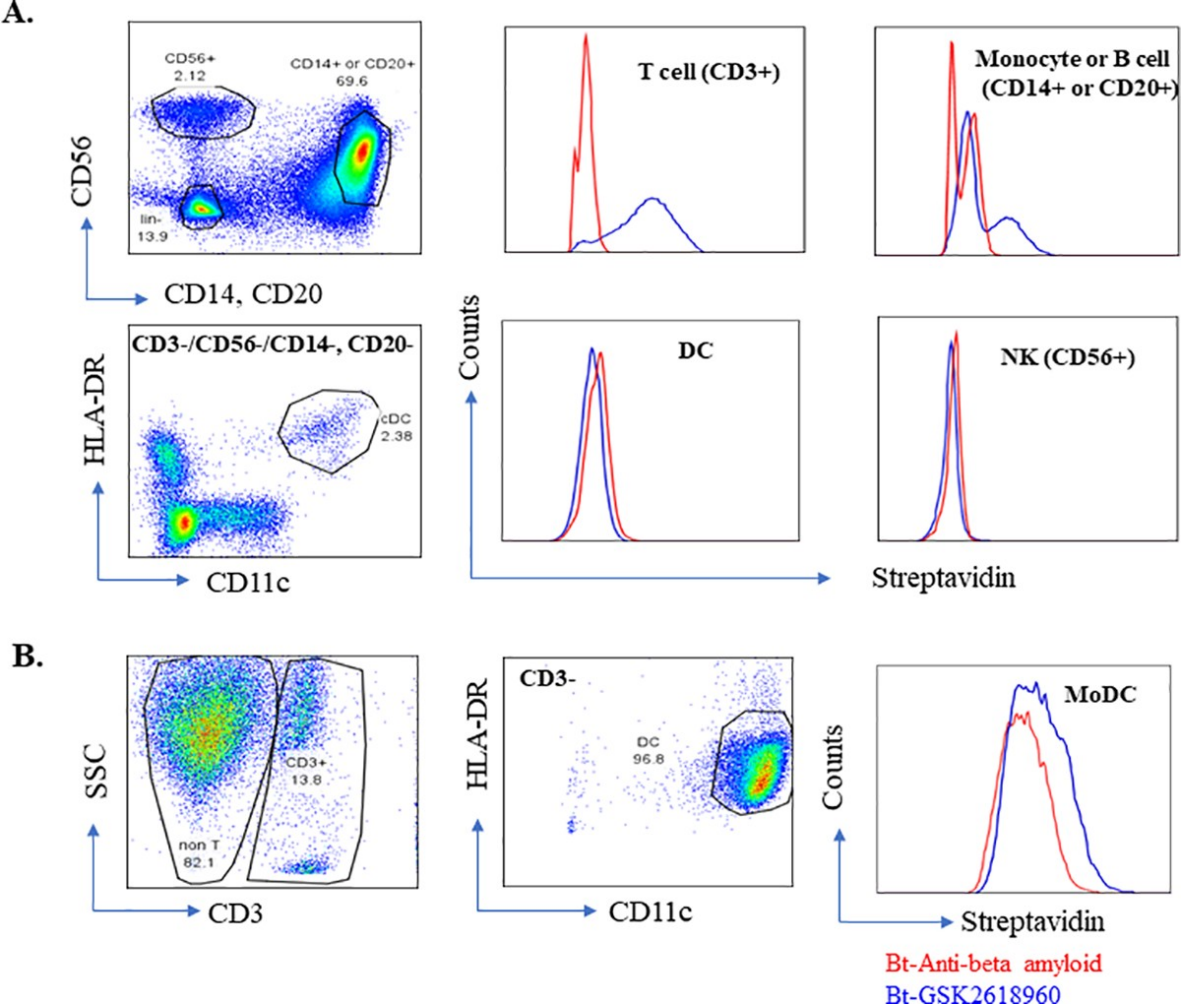
GSK2618960 binds T cells, Monocytes, but not DC in fresh PBMC; It binds *ex vivo* generated DC. (A). GSK2618960 binding evaluation with fresh PBMC. Compared to Fc mutation control antibody–anti beta amyloid (red), GSK2618960 (blue) binds CD3^+^ T cell, Monocyte (CD14^+^) or B cell (CD20^+^), but not NK (CD56^+^), and DC (lin-, HLA DR^+^, CD11c^+^). (B). Representative graphs of GSK2618960 binding on ex vivo generated DC.

Since GSK2618960 binds to a receptor (i.e., IL-7Rα) expressed on immune cells (e.g., DCs and T cells), a robust immunogenicity response as described above can be driven either by the sequence-related antigenicity properties of the drug or potentially augmented through receptor-mediated activity. To explore the latter possibility, the binding of GSK2618960 to different subsets of immune cells was evaluated. Freshly prepared PBMCs were stained with a cocktail of antibodies (anti-CD3, CD56, CD14, CD20, CD11c, and HLA-DR). After washing, samples were further stained with either biotin-labeled GSK2618960 or biotin-labeled Fc control antibody (anti-BAM mAb). The binding of biotin labeled antibody to cells was detected by streptavidin (PE). As shown in Fig 5A, GSK2618960 binds to CD3^+^ T cells as expected due to expression of IL-7Rα on T cells [21–23]. The binding of GSK2618960 to CD3^+^ T cells was previously demonstrated by a full receptor occupancy following administration of GSK2618960 from this phase I study [9]. GSK2618960 was also observed to bind on CD14^+^ monocytes as shown in Fig 5A. CD14^+^ monocytes and CD20^+^ B cells were included together because of same fluorochrome being used for the anti-CD14 and anti-CD20 antibodies. Since IL-7Rα is not known to be expressed on mature B cells [24–26], the GSK2618960 positive subset seen (Fig 5A) was likely only the CD14^+^ cell population. Additionally, GSK2816960 did not bind to NK cells (subset of CD3^-^ and CD56^+^), nor was it found to bind to DCs (CD3^-^, CD56^-^, CD14^-^/CD20^-^ but CD11c/HLA-DR double positive) (Fig 5A). Because there are reports that IL-7Rα expression can be induced in *ex vivo* cultured DCs [26–28], we evaluated whether GSK2618960 binds to ex *vivo* generated monocyte-derived DCs. Isolated CD14^+^ cells were cultured in serum free medium supplemented with GM-CSF and IL-4 for 5 days, and the monocyte derived DC (moDC) cells were harvested for staining with the same surface marker cocktail (see above) and biotin-labeled GSK2618960 or biotin labeled Fc-control antibody (anti-BAM mAb). A clear shift of biotin-labeled GSK2618960 compared to Fc-control antibody indicated the binding of GSK2618960 on MoDC (CD3^-^, CD11c and HLA-DR double positive) (Fig 5B). This shift was observed in MoDC generated from multiple donors (S2 Fig).

**Fig 6:**
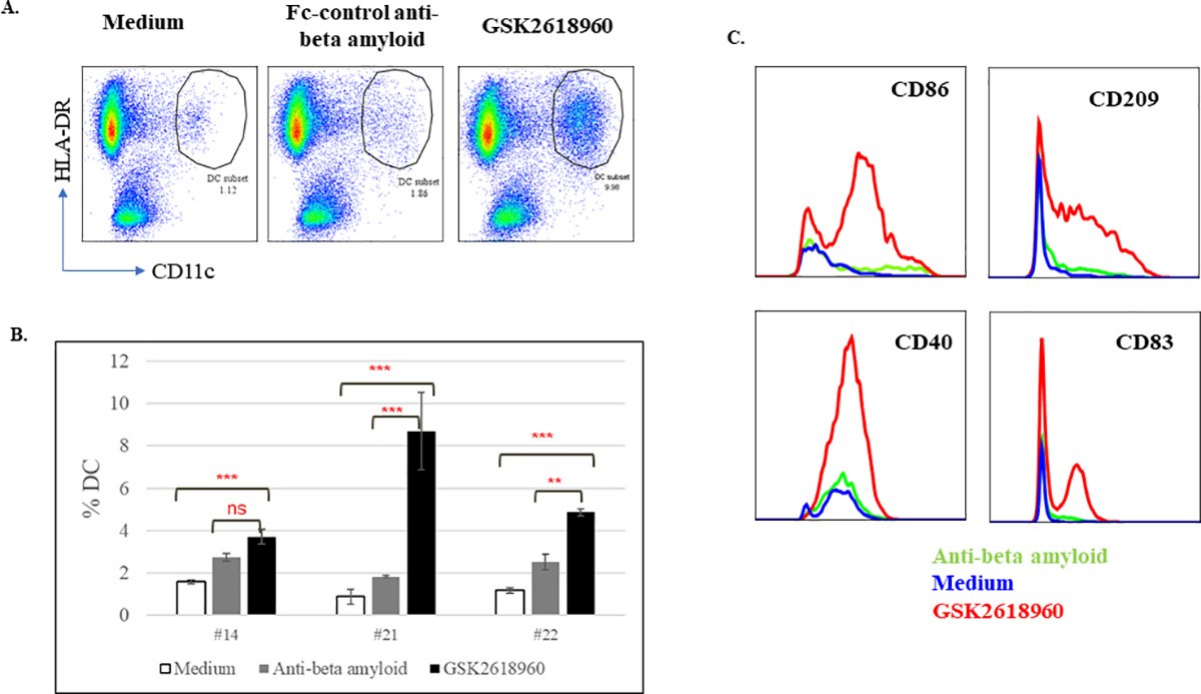
Phenotypic evaluation of *ex vivo* GSK2618960-treated PBMC. PBMC from healthy donors were treated with either Fc control anti-beta amyloid antibody or GSK2618960 for 7 days. (A). Increased DC subsets (CD3^-^, HLA-DR^+^, CD11c^+^) in GSK2618960 treatment. (B). Average % DC subset in medium, Fc control or GSK2618960 treatment from three healthy donors (error bars represent the standard deviation of replicate treatment). Statistical comparison test results: ‘ns’ = not statistically significant (p-value ≥ 0.050); ‘*’ = p-value < 0.050; ‘**’ = p-value < 0.010; ‘***’ = p-value < 0.001. (C). Increased expression of CD86, CD209, CD40, and CD83 on DCs in GSK2618960 treatment.

It was shown above that *ex vivo* treatment of GSK2618960 could stimulate CD4^+^ T cell proliferation in PBMC samples collected from clinical study subjects. The limited cell numbers collected from study subjects did not allow for a detailed phenotypic evaluation beyond examining CD4^+^ T cell proliferation by CTV dilution. To further investigate the cellular phenotypes, we collected blood and isolated PBMCs from healthy, GSK2618960-naïve donors. The PBMCs from these healthy donors were treated with GSK2618960 or the Fc-control antibody (anti-β amyloid, an isotype control antibody with the same Fc disabling mutation as GSK2618960) for 7 days in a combination of solution and immobilized format (i.e., dry coating) and then DC surface and activation markers were evaluated simultaneously. Compared to cell culture medium control or Fc-control antibody treatment, an increase of a population with DC phenotype (CD3^-^, CD11c and HLA DR double positive subset) was observed with GSK2618960 treatment. Representative flow cytometry plots are shown in Fig 6A. The increases of DC subset were observed in all three evaluated donors, the average % DC subset from replicate wells for the three treatment conditions are shown in Fig 6B. The increases of DC subset in the treatment of GSK2618960 were significant in all three donors compared to medium control, and increases were significant compared to Fc control antibody in two out of the three donors (Fig 6B). Moreover, the DC subset from GSK2618960 treatment showed dramatic increases of CD83, CD86, CD40 and CD209 expression compared to medium and Fc antibody controls, suggesting an enhanced DC differentiation and activation in GSK2618960 treatment (Fig 6C). Treatment of the Fc-control mAb showed some increase on the percentage of DC subset relative to medium control; however, the expression of DC surface markers (CD83, CD86, CD40, and CD209) remained at similar levels compared to the medium control. Therefore, the observed impact on DC subsets by GSK2618960 was likely attributable to the antibody functionality related to its complementarity determining regions (CDRs) rather than the Fc domain binding.

## 4. Discussion

Here we report that GSK2618960, a humanized anti-IL7Rα therapeutic monoclonal antibody was found to have a high incidence of immunogenicity in clinical study 200902. Single doses of GSK2618960 in healthy subjects elicited up to 100% ADA incidence with relatively high titers, and neutralizing activity, and the durations of the ADA responses were persistent. Product-related factors, such as aggregates appeared to not be a concern for GSK2618960 as aggregates from the clinical material were within the established product quality attributes specifications (<0.5% aggregates) (data not shown). Patient-related risk factors such as immune status or co-medications appeared to be irrelevant since the subjects enrolled were healthy volunteers. Often, the immunogenicity of humanized antibodies is associated with the unique (nonhuman) sequences in the complementarity determining regions (CDRs) [3]. For GSK2618960, a humanized antibody with CDRs of murine origin, the immunogenicity risk associated with the primary sequence of the CDRs has been evaluated using *in silico* tools to predict potential MHC class II T cell binding epitopes. The *in silico* sequence analysis indicated a low immunogenicity risk for GSK2618960 with risk scores compatible to two benchmark monoclonal antibodies with known low clinical immunogenicity response (e.g., Avastin® and Xolair®). Thus, the *in silico* sequence analysis suggested that the primary sequence of murine CDR sequence was not likely the main cause for the robust clinical immunogenicity response.

Therapeutic mAbs against cell surface receptors, particularly those expressed on immune cells, are generally considered to have an elevated risk for immunogenicity compared to mAbs against soluble targets [3]. Similar to the clinical immunogenicity incidence of GSK2618960 described above, another humanized anti-IL-7Rα (CD127) mAb, PF-06342674, in clinical development also elicited high incidence of ADA in two separate clinical trials [4, 29]. Collectively, the evidence we have presented in this report supports the notion that receptor-mediated biological activity is related to the strong clinical immunogenicity observed for GSK2618960. The reasons for higher risk of immunogenicity for antibodies targeting cell surface receptors are not completely understood. One hypothesis is that binding of the biotherapeutic to the cell surface receptor may enhance internalization and subsequent processing and presentation by antigen presenting cells [30, 31]. GSK2618960 selectively binds to IL-7Rα (CD127), a subunit of receptors for IL-7 and TSLP. Consistent with the reported IL-7Rα expression in T cells and myeloid cells, GSK2618960 was found to bind to CD3^+^ T cells and monocytes, but not NK cells (Fig 5A). Furthermore, we have shown that GSK2618960 did not bind to native DCs (using uncultured PBMCs), but it did bind to *ex vivo* monocyte-derived DCs (Fig 5B). This observation is in agreement with reports from other research groups, that IL-7Rα protein is not readily detectable in *ex vivo* isolated mDC while it can be detected in *in vitro* activated DCs [27, 32].

Given our observation that GSK2618960 binds to monocyte-derived DC (MoDC), we wanted to see whether GSK2618960 had any impact on DC phenotype or activation. Indeed, *ex vivo* treatment of PBMCs with GSK2618960 induced a noticeable increase in a subset with DC phenotypic markers (e.g., CD3^-^, HLA-DR^+^, and CD11c^+^) compared to Fc-control antibody (anti-BAM mAb) or cell culture medium (Fig 6). Moreover, the DC subset treated with GSK2618960 showed enhanced activation, which was evident by the increased expression in CD83, CD86, CD40, and CD209 compared to the controls (i.e., Fc control antibody or medium). The increases of DC subpopulations and enhanced DC activation in the treatment of GSK2618960 suggest overall enhancement of DC functionality with GSK2618960 treatment. This elevated DC functionality may be related to the strong *in vivo* clinical immunogenicity response observed with GSK2618960. Whether the observed increase of DC subsets and expanded DC activation are consequences of enhanced antigen internalization, processing, and presentation facilitated by binding of GSK2618960 to IL-7R expressed on DCs or a direct signaling mechanism from receptor engagement via binding of GSK2618960 on DCs remains to be elucidated.

Evidence exists from clinical studies that GSK2618960 exhibited a degree of paradoxical agonistic potential in *ex vivo* assays using PBMCs from study subjects [9]. It was noted that addition of GSK2618960 to pre-dose PBMC samples from study subjects (samples were not treated with IL-7) induced the phosphorylation of STAT5 in CD3^+^/CD4^+^ T cells, indicating receptor agonism by GSK2618960. Although the degree of pSTAT5 induction was small (i.e., an average increase of ~7% compared to the control response, which was IL-7 treatment alone), it was observed in 17 of 18 PBMC samples [9]. Low levels of STAT5 phosphorylation following GSK2618960 treatment of whole blood from healthy donors were observed in an *ex vivo* assay, suggesting potential stimulating activity of IL-7R (GSK unpublished data). The binding of GSK2618960 to *ex vivo* monocyte-derived DC presented in this report and the agonist activity potential that was previously reported for GSK2618960, provide insight into a potential mechanism for the observed of DC activation and CD4^+^ T cell stimulation potential of GSK2618960 under the *ex vivo* treatment conditions. Consequently, these observations may provide evidence and, in part, explain the robust humoral immune response against GSK2618960 observed in the clinical study.

The detection of GSK2618960-specific memory B cells in treated subjects indicated the development of an ADA response that included immune memory activity against GSK2618960 following single dose in study subjects. The frequency of the specific memory B cells from positive response subjects seemed higher in 2 mg/kg cohort compared to 0.6 mg/kg cohort (16.9 v.s. 5.2 ASC per million PBMC) (S1 Table). Evidence of a semi-quantitative correlation between ADA titer and memory B cell activity was found on Day 169. Based on this data, it is hypothesized that memory B cell activity may be responsible for the persistent nature of the ADA response. The implication of this ADA-specific memory B activity in ADA development and clinical consequences for repeat dosing is unclear. Memory B cells are programmed to respond rapidly to antigen exposure, proliferating and differentiating into antibody-secreting cells quickly after detecting antigen [33–35]. In principle, the development of GSK2618960-specific memory B activity indicates the possibility of a rapid increase of ADAs in case of subsequent dosing, leading to an increased risk for altered PK, PD, or emergent safety signals. The observed ADA-specific memory B activity also suggests involvement of helper T cells in the GSK2618960-specific immune response. To this end, we demonstrated that treatment of the subject’s PBMCs with GSK2618960 resulted in strong CD4^+^ T cell stimulation compared to the control anti-RSV mAb, using an *ex vivo* CD4^+^ T cell proliferation method. Furthermore, the results from this assay also revealed a dose-dependent increase in CD4^+^ T cell stimulation by GSK2618960. These data support the role of helper T cells in the immune response to GSK2618960. While it would be important to determine whether GSK2618960-treated subjects developed memory T cells, the *ex vivo* CD4 T^+^ cell proliferation assay was not equipped to differentiate between memory and naïve CD4^+^ T cell responses. Of note, the CD4^+^ T cell proliferation observed in treatment naïve (i.e., placebo) subjects suggested a *de novo* response to GSK2618960 treatment. Finally, the strong *ex vivo* CD4^+^ T cell stimulation potential correlated well with the robust clinical ADA incidence. Our data provide further support for the utility of *ex vivo* CD4^+^ T cell proliferation assays for clinical immunogenicity prediction.

In summary, we report a robust immunogenicity response following the administration of a single dose of GSK2618960 in healthy human subjects. Our data supports the notion that an unintended receptor-mediated agonistic activity (an unfortunate property of a receptor blocking antibody) was the main risk factor contributing to the robust clinical immunogenicity response observed for GSK2618960. IL-7Rα, and its signaling pathway, remains a potentially important target for both cancer and autoimmune diseases [36–40]. The findings described in this report might be helpful for further therapeutic development of anti-IL7-Rα biotherapeutics. The *in vitro* methods applied in this investigation are relevant for characterizing immunogenicity responses and identifying potential immunogenicity risks for biotherapeutics that target cellular receptors. Furthermore, *in vitro* methods to assess immunogenicity potential for biotherapeutics can serve as valuable tools to aid in de-risking immunogenicity during the lead development stage.

## Supporting information

S1 Fig CONSORT flow diagram. * Safety population was used fog assessment of safety, PD/biomarkers and immunogenicity.
(TIF)

S2 Fig (TIF)

S3 Fig (TIF)

S1 Table (TIF)
