# Supplementary figures and images for: Characterization of the robust humoral immune response to GSK2618960, a humanized anti-IL-7 receptor monoclonal antibody, observed in healthy subjects in a Phase 1 study
Source: PLoS One. 2021 Mar 23;16(3):e0249049. doi: 10.1371/journal.pone.0249049 (PMC7987154; doi:10.1371/journal.pone.0249049)

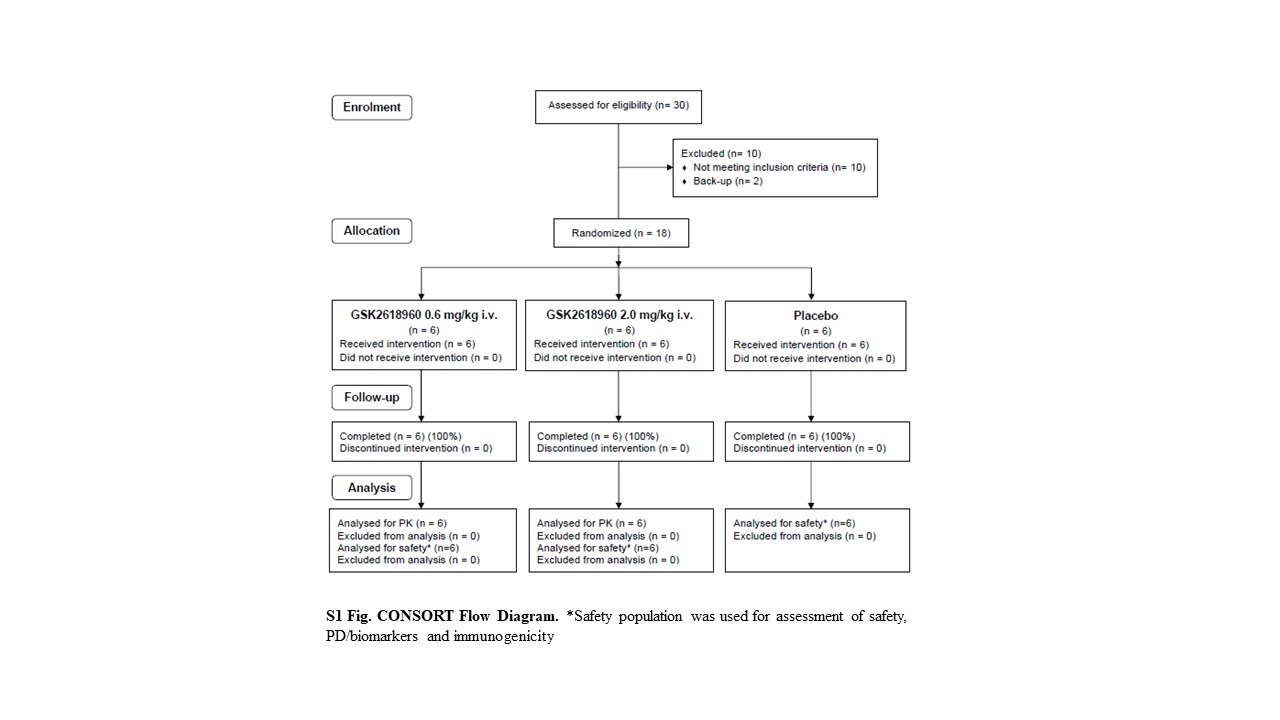

Supplement: S1 Fig — * Safety population was used fog assessment of safety, PD/biomarkers and immunogenicity. (TIF) [file pone.0249049.s001.tif]

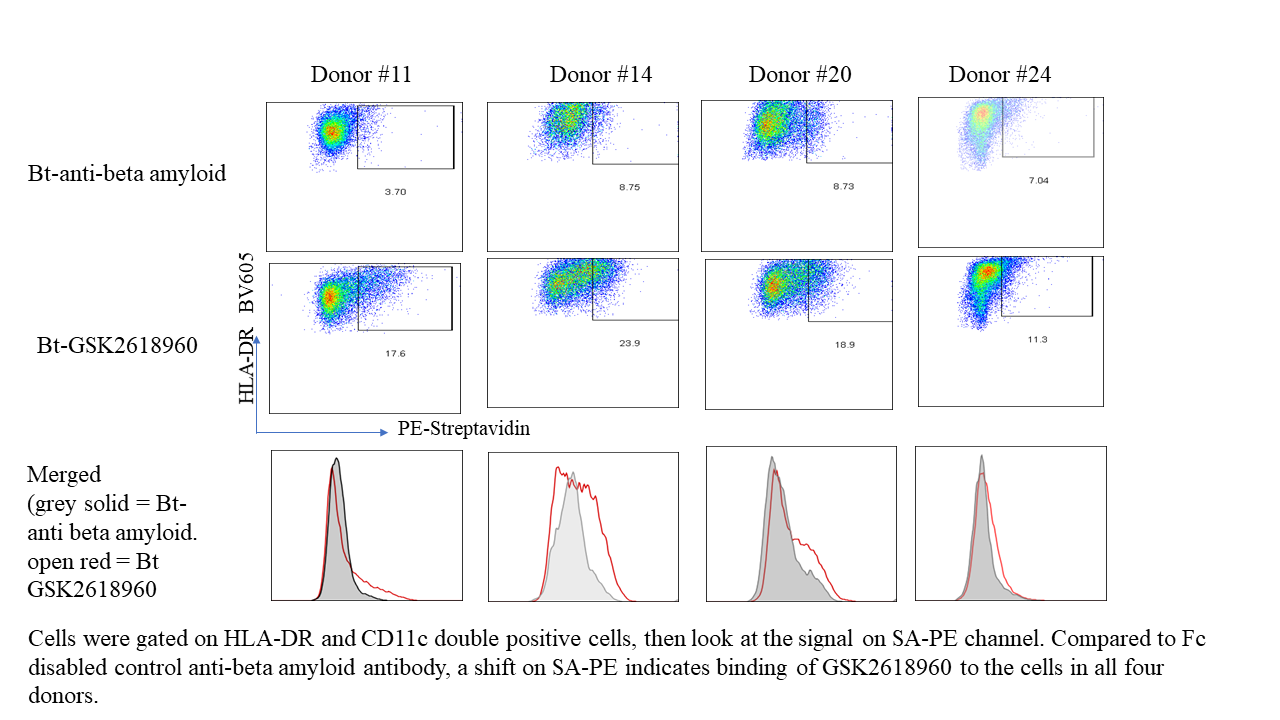

Supplement: S2 Fig — (TIF) [file pone.0249049.s002.tif]

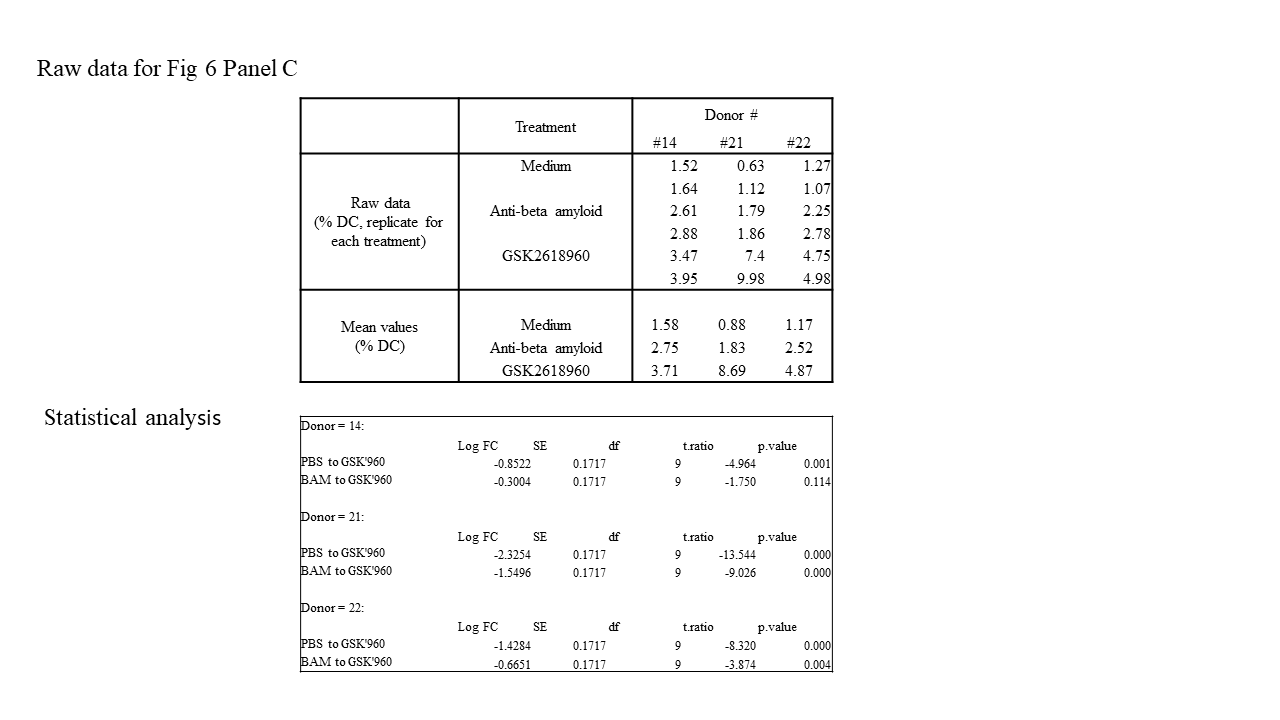

Supplement: S3 Fig — (TIF) [file pone.0249049.s003.tif]

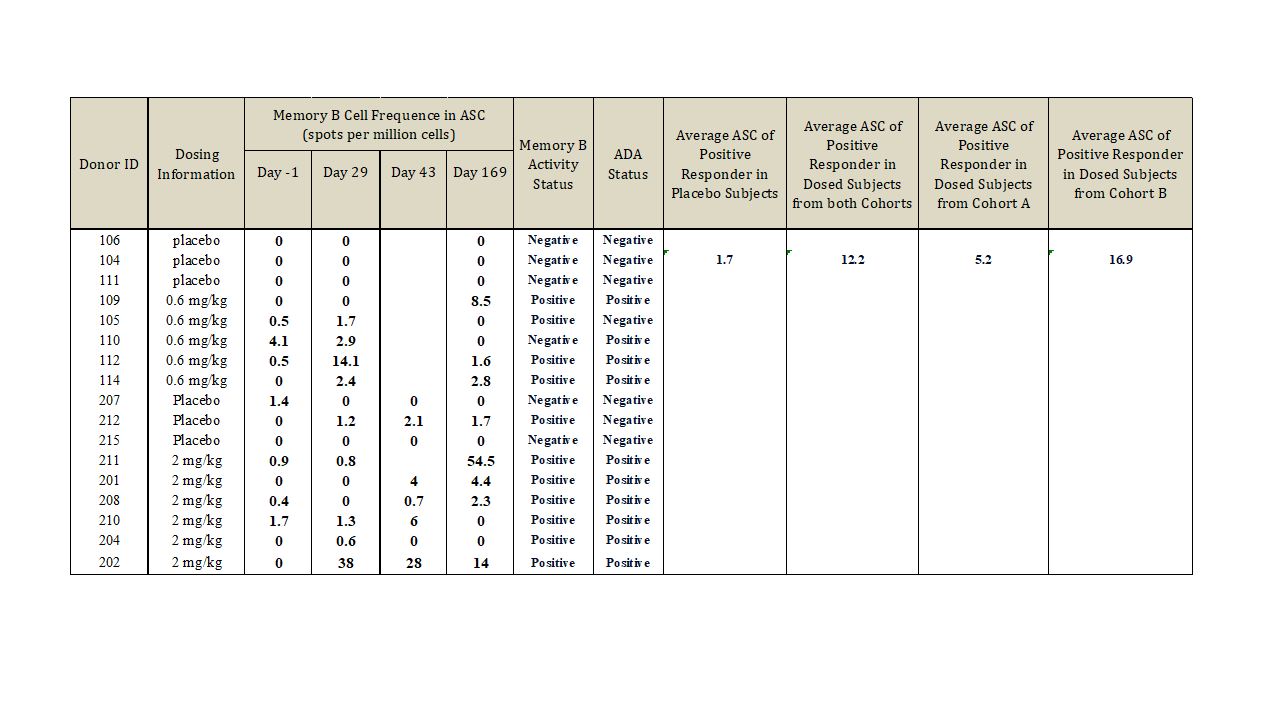

Supplement: S1 Table — (TIF) [file pone.0249049.s004.tif]
